# (*E*)-Ethyl 2-benzoyl-4-(naphthalen-2-yl)-4-oxobut-2-enoate

**DOI:** 10.1107/S1600536811018745

**Published:** 2011-05-25

**Authors:** Liuming Wu, Cong Deng, Yan Yang

**Affiliations:** a Key Laboratory of Pesticide and Chemical Biology of the Ministry of Education, College of Chemistry, Central China Normal University, Wuhan 430079, People’s Republic of China

## Abstract

The title compound, C_23_H_18_O_4_, is a 1,4-enedione compound which contains a naphthalene ring and a benzene ring. The dihedral angle between the ring systems is 74.9 (2)°. In the crystal, the mol­ecules form π–π stacking inter­actions between naphthalene rings of inversion-related mol­ecules, with an inter­planar spacing of 3.499 (2) Å.

## Related literature

For the preparation of the title compound, see: Gao *et al.* (2010 ▶). For related structures, see: Prakash *et al.* (2005 ▶); Raj *et al.* (1996 ▶). 
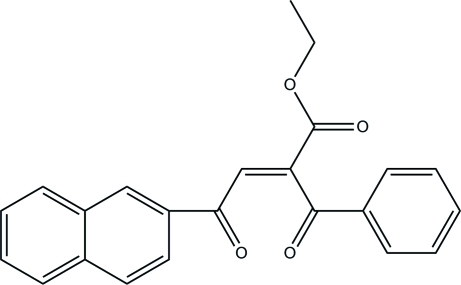


## Experimental

### 

#### Crystal data

C_23_H_18_O_4_
*M* *_r_* = 358.37
Triclinic, 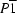

*a* = 7.8571 (13) Å
*b* = 9.6157 (16) Å
*c* = 13.934 (2) Å
α = 99.364 (3)°
β = 105.094 (3)°
γ = 110.648 (3)°
*V* = 912.6 (3) Å^3^
*Z* = 2
Mo *K*α radiation
μ = 0.09 mm^−1^
*T* = 298 K
0.16 × 0.12 × 0.10 mm

#### Data collection

Bruker SMART CCD area-detector diffractometer
5661 measured reflections
3355 independent reflections
2818 reflections with *I* > 2σ(*I*)
*R*_int_ = 0.065

#### Refinement

*R*[*F* ^2^ > 2σ(*F* ^2^)] = 0.054
*wR*(*F* ^2^) = 0.149
*S* = 1.05
3355 reflections
245 parameters
H-atom parameters constrained
Δρ_max_ = 0.18 e Å^−3^
Δρ_min_ = −0.19 e Å^−3^

### 

Data collection: *SMART* (Bruker, 2001 ▶); cell refinement: *SAINT* (Bruker, 2001 ▶); data reduction: *SAINT*; program(s) used to solve structure: *SHELXS97* (Sheldrick, 2008 ▶); program(s) used to refine structure: *SHELXL97* (Sheldrick, 2008 ▶); molecular graphics: *PLATON* (Spek, 2009 ▶); software used to prepare material for publication: *PLATON*, *SHELXL97* and *publCIF* (Westrip, 2010 ▶).

## Supplementary Material

Crystal structure: contains datablocks I, global. DOI: 10.1107/S1600536811018745/pk2318sup1.cif

Structure factors: contains datablocks I. DOI: 10.1107/S1600536811018745/pk2318Isup2.hkl

Supplementary material file. DOI: 10.1107/S1600536811018745/pk2318Isup3.cml

Additional supplementary materials:  crystallographic information; 3D view; checkCIF report

## Figures and Tables

**Fig. 1.**
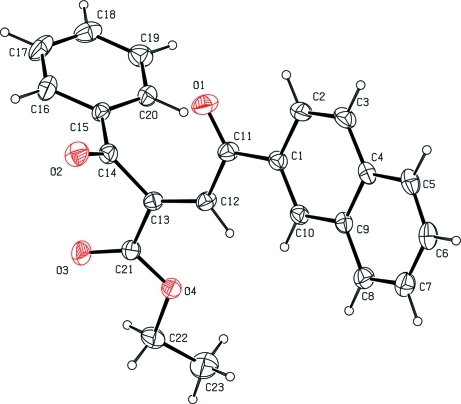
A view of the compound with displacement ellipsoids drawn at the 30% probability level.

## supplementary crystallographic information

### Comment

The 1,4-enedione framework is frequently found in bioactive natural products and medicinal compounds.  In addition, by virtue of their multifunctional composition, 1,4-enediones could serve as versatile precursors for heterocycle synthesis, Diels-Alder cycloaddition, as well as many other useful transformations. We report here the crystal structure of the title compound (Fig. 1). The crystal packing exhibits offset π-π stacking interactions.

### Experimental

The title compound was synthesized according to the reported literature (Gao *et al.*, 2010).  Crystals suitable for X-ray diffraction were grown by slow evaporation of a ethyl acetate-hexane (2:1) solution of the title compound at 293 K.

### Refinement

All H atoms were positioned in geometrically idealized positions and constrained to ride on their parent atoms, with C—H distances in the range 0.93–0.97 Å and *U*_iso_(H) = 1.2*U*_eq_(C) or *U*_iso_(H) = 1.5*U*_eq_(C_Me_).

### Crystal data

**Table:**

C_23_H_18_O_4_	*Z* = 2
*M**_r_* = 358.37	*F*(000) = 376
Triclinic, *P*1	*D*_x_ = 1.304 Mg m^−^^3^
Hall symbol: -P 1	Mo *K*α radiation, λ = 0.71073 Å
*a* = 7.8571 (13) Å	Cell parameters from 2846 reflections
*b* = 9.6157 (16) Å	θ = 2.4–27.7°
*c* = 13.934 (2) Å	µ = 0.09 mm^−^^1^
α = 99.364 (3)°	*T* = 298 K
β = 105.094 (3)°	Block, colorless
γ = 110.648 (3)°	0.16 × 0.12 × 0.10 mm
*V* = 912.6 (3) Å^3^	

### Data collection

**Table:**

Bruker SMART CCD area-detector diffractometer	2818 reflections with *I* > 2σ(*I*)
Radiation source: fine-focus sealed tube	*R*_int_ = 0.065
graphite	θ_max_ = 25.5°, θ_min_ = 2.4°
φ and ω scans	*h* = −8→9
5661 measured reflections	*k* = −11→9
3355 independent reflections	*l* = −16→16

### Refinement

**Table:**

Refinement on *F*^2^	Primary atom site location: structure-invariant direct methods
Least-squares matrix: full	Secondary atom site location: difference Fourier map
*R*[*F*^2^ > 2σ(*F*^2^)] = 0.054	Hydrogen site location: inferred from neighbouring sites
*wR*(*F*^2^) = 0.149	H-atom parameters constrained
*S* = 1.05	*w* = 1/[σ^2^(*F*_o_^2^) + (0.0672*P*)^2^ + 0.1681*P*] where *P* = (*F*_o_^2^ + 2*F*_c_^2^)/3
3355 reflections	(Δ/σ)_max_ < 0.001
245 parameters	Δρ_max_ = 0.18 e Å^−^^3^
0 restraints	Δρ_min_ = −0.19 e Å^−^^3^

### Special details

**Table:**

Geometry. All e.s.d.'s (except the e.s.d. in the dihedral angle between two l.s. planes) are estimated using the full covariance matrix. The cell e.s.d.'s are taken into account individually in the estimation of e.s.d.'s in distances, angles and torsion angles; correlations between e.s.d.'s in cell parameters are only used when they are defined by crystal symmetry. An approximate (isotropic) treatment of cell e.s.d.'s is used for estimating e.s.d.'s involving l.s. planes.
Refinement. Refinement of *F*^2^ against ALL reflections. The weighted *R*-factor *wR* and goodness of fit *S* are based on *F*^2^, conventional *R*-factors *R* are based on *F*, with *F* set to zero for negative *F*^2^. The threshold expression of *F*^2^ > σ(*F*^2^) is used only for calculating *R*-factors(gt) *etc*. and is not relevant to the choice of reflections for refinement. *R*-factors based on *F*^2^ are statistically about twice as large as those based on *F*, and *R*- factors based on ALL data will be even larger.

### Fractional atomic coordinates and isotropic or equivalent isotropic displacement parameters (Å^2^)

**Table:**

	*x*	*y*	*z*	*U*_iso_*/*U*_eq_	
C1	0.7525 (2)	0.3380 (2)	0.02603 (13)	0.0480 (4)	
C2	0.6361 (3)	0.2384 (2)	−0.07533 (14)	0.0594 (5)	
H2	0.6065	0.1330	−0.0892	0.071*	
C3	0.5681 (3)	0.2958 (2)	−0.15181 (14)	0.0634 (5)	
H3	0.4969	0.2295	−0.2183	0.076*	
C4	0.6024 (3)	0.4539 (2)	−0.13326 (13)	0.0526 (4)	
C5	0.5236 (3)	0.5168 (3)	−0.20967 (15)	0.0644 (5)	
H5	0.4507	0.4533	−0.2768	0.077*	
C6	0.5524 (3)	0.6673 (3)	−0.18670 (17)	0.0666 (6)	
H6	0.4974	0.7056	−0.2377	0.080*	
C7	0.6642 (3)	0.7654 (3)	−0.08709 (17)	0.0673 (6)	
H7	0.6839	0.8689	−0.0721	0.081*	
C8	0.7445 (3)	0.7107 (2)	−0.01181 (15)	0.0602 (5)	
H8	0.8192	0.7776	0.0542	0.072*	
C9	0.7167 (2)	0.5546 (2)	−0.03195 (13)	0.0479 (4)	
C10	0.7908 (3)	0.4926 (2)	0.04557 (13)	0.0486 (4)	
H10	0.8677	0.5582	0.1117	0.058*	
C11	0.8160 (3)	0.2679 (2)	0.10791 (14)	0.0529 (4)	
C12	0.9526 (3)	0.3702 (2)	0.21270 (14)	0.0508 (4)	
H12	1.0266	0.4736	0.2201	0.061*	
C13	0.9720 (2)	0.31813 (19)	0.29631 (13)	0.0458 (4)	
C14	0.8554 (3)	0.1555 (2)	0.29805 (14)	0.0502 (4)	
C15	0.9508 (2)	0.04721 (18)	0.29869 (13)	0.0466 (4)	
C16	0.9100 (3)	−0.0649 (2)	0.35035 (17)	0.0670 (6)	
H16	0.8236	−0.0716	0.3859	0.080*	
C17	0.9978 (4)	−0.1679 (3)	0.3492 (2)	0.0811 (7)	
H17	0.9701	−0.2430	0.3841	0.097*	
C18	1.1244 (4)	−0.1585 (2)	0.29701 (19)	0.0737 (6)	
H18	1.1812	−0.2285	0.2954	0.088*	
C19	1.1676 (3)	−0.0474 (3)	0.24748 (18)	0.0707 (6)	
H19	1.2550	−0.0409	0.2126	0.085*	
C20	1.0828 (3)	0.0558 (2)	0.24846 (15)	0.0562 (5)	
H20	1.1147	0.1322	0.2149	0.067*	
C21	1.1107 (3)	0.4188 (2)	0.40120 (13)	0.0500 (4)	
C22	1.3699 (3)	0.6602 (3)	0.49986 (16)	0.0761 (6)	
H22A	1.4252	0.6045	0.5412	0.091*	
H22B	1.3052	0.7056	0.5372	0.091*	
C23	1.5227 (4)	0.7817 (3)	0.4819 (2)	0.0995 (9)	
H23A	1.5909	0.7365	0.4485	0.149*	
H23B	1.6114	0.8551	0.5470	0.149*	
H23C	1.4664	0.8333	0.4386	0.149*	
O1	0.7583 (2)	0.12815 (15)	0.09194 (11)	0.0762 (5)	
O2	0.6987 (2)	0.12270 (17)	0.30777 (13)	0.0743 (4)	
O3	1.1113 (3)	0.37690 (18)	0.47767 (11)	0.0809 (5)	
O4	1.23194 (19)	0.55455 (14)	0.40043 (9)	0.0607 (4)	

### Atomic displacement parameters (Å^2^)

**Table:**

	*U*^11^	*U*^22^	*U*^33^	*U*^12^	*U*^13^	*U*^23^
C1	0.0466 (9)	0.0505 (10)	0.0416 (9)	0.0165 (8)	0.0136 (7)	0.0100 (7)
C2	0.0688 (12)	0.0530 (11)	0.0468 (10)	0.0222 (9)	0.0148 (9)	0.0046 (8)
C3	0.0684 (13)	0.0687 (13)	0.0375 (10)	0.0227 (10)	0.0092 (9)	0.0027 (9)
C4	0.0498 (10)	0.0692 (12)	0.0398 (9)	0.0226 (9)	0.0194 (8)	0.0162 (8)
C5	0.0597 (12)	0.0911 (16)	0.0425 (10)	0.0295 (11)	0.0167 (9)	0.0247 (10)
C6	0.0641 (12)	0.0915 (16)	0.0609 (12)	0.0379 (12)	0.0272 (10)	0.0422 (12)
C7	0.0742 (14)	0.0692 (13)	0.0709 (14)	0.0330 (11)	0.0309 (11)	0.0340 (11)
C8	0.0648 (12)	0.0586 (11)	0.0517 (11)	0.0216 (9)	0.0159 (9)	0.0179 (9)
C9	0.0431 (9)	0.0567 (10)	0.0424 (9)	0.0166 (8)	0.0168 (7)	0.0160 (8)
C10	0.0474 (9)	0.0519 (10)	0.0365 (8)	0.0142 (8)	0.0098 (7)	0.0090 (7)
C11	0.0521 (10)	0.0458 (10)	0.0518 (10)	0.0169 (8)	0.0099 (8)	0.0115 (8)
C12	0.0506 (10)	0.0422 (9)	0.0497 (10)	0.0143 (8)	0.0088 (8)	0.0129 (8)
C13	0.0459 (9)	0.0436 (9)	0.0483 (9)	0.0203 (7)	0.0144 (7)	0.0130 (7)
C14	0.0471 (10)	0.0506 (10)	0.0478 (10)	0.0138 (8)	0.0173 (8)	0.0137 (8)
C15	0.0460 (9)	0.0398 (9)	0.0421 (9)	0.0088 (7)	0.0087 (7)	0.0126 (7)
C16	0.0611 (12)	0.0646 (12)	0.0654 (13)	0.0114 (10)	0.0177 (10)	0.0327 (10)
C17	0.0806 (16)	0.0546 (12)	0.0858 (16)	0.0134 (11)	0.0011 (13)	0.0409 (12)
C18	0.0717 (14)	0.0504 (12)	0.0797 (15)	0.0260 (10)	−0.0012 (12)	0.0122 (11)
C19	0.0788 (15)	0.0704 (14)	0.0694 (13)	0.0409 (12)	0.0239 (11)	0.0163 (11)
C20	0.0666 (12)	0.0527 (10)	0.0564 (11)	0.0274 (9)	0.0244 (9)	0.0224 (9)
C21	0.0552 (10)	0.0496 (10)	0.0468 (10)	0.0230 (8)	0.0165 (8)	0.0164 (8)
C22	0.0786 (15)	0.0692 (14)	0.0457 (11)	0.0092 (12)	0.0082 (10)	−0.0021 (10)
C23	0.0779 (17)	0.0856 (18)	0.0757 (16)	−0.0030 (14)	−0.0046 (13)	0.0066 (13)
O1	0.0878 (11)	0.0466 (8)	0.0652 (9)	0.0192 (7)	−0.0030 (8)	0.0097 (7)
O2	0.0568 (9)	0.0716 (9)	0.0978 (11)	0.0209 (7)	0.0394 (8)	0.0244 (8)
O3	0.1044 (12)	0.0704 (10)	0.0491 (8)	0.0171 (9)	0.0188 (8)	0.0249 (7)
O4	0.0654 (8)	0.0528 (8)	0.0406 (7)	0.0071 (6)	0.0084 (6)	0.0092 (6)

### Geometric parameters (Å, °)

**Table:**

C1—C10	1.373 (2)	C13—C14	1.517 (2)
C1—C2	1.420 (3)	C14—O2	1.208 (2)
C1—C11	1.482 (2)	C14—C15	1.482 (3)
C2—C3	1.352 (3)	C15—C20	1.381 (3)
C2—H2	0.9300	C15—C16	1.383 (3)
C3—C4	1.413 (3)	C16—C17	1.392 (3)
C3—H3	0.9300	C16—H16	0.9300
C4—C5	1.419 (3)	C17—C18	1.365 (4)
C4—C9	1.421 (3)	C17—H17	0.9300
C5—C6	1.352 (3)	C18—C19	1.356 (3)
C5—H5	0.9300	C18—H18	0.9300
C6—C7	1.394 (3)	C19—C20	1.377 (3)
C6—H6	0.9300	C19—H19	0.9300
C7—C8	1.358 (3)	C20—H20	0.9300
C7—H7	0.9300	C21—O3	1.198 (2)
C8—C9	1.406 (3)	C21—O4	1.321 (2)
C8—H8	0.9300	C22—O4	1.453 (2)
C9—C10	1.410 (2)	C22—C23	1.459 (3)
C10—H10	0.9300	C22—H22A	0.9700
C11—O1	1.217 (2)	C22—H22B	0.9700
C11—C12	1.491 (2)	C23—H23A	0.9600
C12—C13	1.335 (2)	C23—H23B	0.9600
C12—H12	0.9300	C23—H23C	0.9600
C13—C21	1.492 (2)		
			
C10—C1—C2	118.91 (16)	C21—C13—C14	112.15 (14)
C10—C1—C11	122.81 (15)	O2—C14—C15	122.53 (16)
C2—C1—C11	118.14 (16)	O2—C14—C13	120.11 (16)
C3—C2—C1	120.47 (18)	C15—C14—C13	117.05 (15)
C3—C2—H2	119.8	C20—C15—C16	118.37 (18)
C1—C2—H2	119.8	C20—C15—C14	121.17 (15)
C2—C3—C4	121.70 (17)	C16—C15—C14	120.46 (17)
C2—C3—H3	119.2	C15—C16—C17	120.2 (2)
C4—C3—H3	119.2	C15—C16—H16	119.9
C3—C4—C5	123.50 (18)	C17—C16—H16	119.9
C3—C4—C9	118.46 (17)	C18—C17—C16	120.1 (2)
C5—C4—C9	118.00 (18)	C18—C17—H17	120.0
C6—C5—C4	121.28 (19)	C16—C17—H17	120.0
C6—C5—H5	119.4	C19—C18—C17	120.2 (2)
C4—C5—H5	119.4	C19—C18—H18	119.9
C5—C6—C7	120.40 (19)	C17—C18—H18	119.9
C5—C6—H6	119.8	C18—C19—C20	120.4 (2)
C7—C6—H6	119.8	C18—C19—H19	119.8
C8—C7—C6	120.4 (2)	C20—C19—H19	119.8
C8—C7—H7	119.8	C19—C20—C15	120.80 (18)
C6—C7—H7	119.8	C19—C20—H20	119.6
C7—C8—C9	121.17 (19)	C15—C20—H20	119.6
C7—C8—H8	119.4	O3—C21—O4	124.25 (17)
C9—C8—H8	119.4	O3—C21—C13	122.09 (17)
C8—C9—C10	122.55 (16)	O4—C21—C13	113.65 (15)
C8—C9—C4	118.77 (17)	O4—C22—C23	108.74 (19)
C10—C9—C4	118.61 (16)	O4—C22—H22A	109.9
C1—C10—C9	121.80 (16)	C23—C22—H22A	109.9
C1—C10—H10	119.1	O4—C22—H22B	109.9
C9—C10—H10	119.1	C23—C22—H22B	109.9
O1—C11—C1	121.54 (17)	H22A—C22—H22B	108.3
O1—C11—C12	118.93 (16)	C22—C23—H23A	109.5
C1—C11—C12	119.53 (15)	C22—C23—H23B	109.5
C13—C12—C11	122.11 (16)	H23A—C23—H23B	109.5
C13—C12—H12	118.9	C22—C23—H23C	109.5
C11—C12—H12	118.9	H23A—C23—H23C	109.5
C12—C13—C21	122.55 (16)	H23B—C23—H23C	109.5
C12—C13—C14	125.27 (16)	C21—O4—C22	116.98 (15)
			
C10—C1—C2—C3	1.5 (3)	C11—C12—C13—C21	178.92 (16)
C11—C1—C2—C3	177.41 (18)	C11—C12—C13—C14	−3.1 (3)
C1—C2—C3—C4	−2.8 (3)	C12—C13—C14—O2	−84.9 (2)
C2—C3—C4—C5	−175.90 (18)	C21—C13—C14—O2	93.3 (2)
C2—C3—C4—C9	2.0 (3)	C12—C13—C14—C15	101.4 (2)
C3—C4—C5—C6	176.54 (19)	C21—C13—C14—C15	−80.41 (19)
C9—C4—C5—C6	−1.4 (3)	O2—C14—C15—C20	153.77 (19)
C4—C5—C6—C7	1.2 (3)	C13—C14—C15—C20	−32.7 (2)
C5—C6—C7—C8	−0.4 (3)	O2—C14—C15—C16	−26.5 (3)
C6—C7—C8—C9	−0.3 (3)	C13—C14—C15—C16	147.00 (18)
C7—C8—C9—C10	−177.10 (18)	C20—C15—C16—C17	−1.3 (3)
C7—C8—C9—C4	0.1 (3)	C14—C15—C16—C17	178.96 (18)
C3—C4—C9—C8	−177.29 (18)	C15—C16—C17—C18	−0.1 (3)
C5—C4—C9—C8	0.7 (3)	C16—C17—C18—C19	1.1 (4)
C3—C4—C9—C10	0.0 (2)	C17—C18—C19—C20	−0.7 (3)
C5—C4—C9—C10	178.01 (16)	C18—C19—C20—C15	−0.8 (3)
C2—C1—C10—C9	0.4 (3)	C16—C15—C20—C19	1.7 (3)
C11—C1—C10—C9	−175.22 (16)	C14—C15—C20—C19	−178.54 (18)
C8—C9—C10—C1	176.00 (17)	C12—C13—C21—O3	170.97 (19)
C4—C9—C10—C1	−1.2 (3)	C14—C13—C21—O3	−7.2 (2)
C10—C1—C11—O1	169.80 (19)	C12—C13—C21—O4	−10.2 (2)
C2—C1—C11—O1	−5.9 (3)	C14—C13—C21—O4	171.62 (15)
C10—C1—C11—C12	−9.7 (3)	O3—C21—O4—C22	−2.0 (3)
C2—C1—C11—C12	174.56 (16)	C13—C21—O4—C22	179.16 (16)
O1—C11—C12—C13	−19.2 (3)	C23—C22—O4—C21	164.2 (2)
C1—C11—C12—C13	160.31 (17)		
